# Validation of an NGS Panel Designed for Detection of Actionable Mutations in Tumors Common in Latin America

**DOI:** 10.3390/jpm11090899

**Published:** 2021-09-08

**Authors:** Mauricio Salvo, Evelin González-Feliú, Jessica Toro, Iván Gallegos, Ignacio Maureira, Nicolás Miranda-González, Olga Barajas, Eva Bustamante, Mónica Ahumada, Alicia Colombo, Ricardo Armisén, Camilo Villamán, Carolina Ibañez, María Loreto Bravo, Verónica Sanhueza, M. Loreto Spencer, Gonzalo de Toro, Erik Morales, Carolina Bizama, Patricia García, Ana María Carrasco, Lorena Gutiérrez, Justo Lorenzo Bermejo, Ricardo A. Verdugo, Katherine Marcelain

**Affiliations:** 1 Department of Basic and Clinical Oncology, Faculty of Medicine, Universidad de Chile, Santiago 8330015, Chile; mauricio.salvo@roche.com (M.S.); evefeliu@gmail.com (E.G.-F.); jessicatoro@med.uchile.cl (J.T.); igallegos@hcuch.cl (I.G.); ignaciomaureira@uchile.cl (I.M.); miranda528491@gmail.com (N.M.-G.); olbeba@gmail.com (O.B.); mahumada@hcuch.cl (M.A.); acolombo@hcuch.cl (A.C.); c.villaman@gmail.com (C.V.); 2 Department of Pathology, Hospital Clínico de la Universidad de Chile, Santiago 8380456, Chile; 3 Department of Medical Technology, Faculty of Medicine, Universidad de Chile, Santiago 8330015, Chile; 4 Department of Internal Medicine, Hospital Clínico Universidad de Chile, Santiago 8380456, Chile; 5 Fundación Arturo López Pérez, Santiago 7500921, Chile; bustamantee@falp.org (E.B.); dracarrasqui@gmail.com (A.M.C.); 6 Center for Genetics and Genomics, Instituto de Ciencias e Innovación en Medicina, Facultad de Medicina Clínica Alemana, Universidad del Desarrollo, Santiago 8320000, Chile; rarmisen@udd.cl; 7 Department of Hematology & Oncology, Faculty of Medicine, Pontificia Universidad Católica de Chile (PUC), Santiago 3580000, Chile; doctoraibanez@gmail.com (C.I.); marialoretobravo@gmail.com (M.L.B.); 8 Department of Pathology, Hospital Padre Hurtado, Santiago 8710022, Chile; verosanhueza@yahoo.com; 9 Department of Pathology, Hospital Clínico Regional Guillermo Grant Benavente, Concepción 4070038, Chile; loretospencer@gmail.com; 10 School of Medical Technology, Universidad Austral de Chile at Puerto Montt, Puerto Montt 5110566, Chile; gdetoro@gmail.com; 11 Department of Pathology, Hospital Regional de Talca, Talca 3460000, Chile; emoralesm@hospitaldetalca.cl; 12 Department of Preclinical Sciences, Faculty of Medicine, Universidad Católica del Maule, Talca 3460000, Chile; 13 Department of Pathology, Faculty of Medicine, Pontificia Universidad Católica de Chile, Santiago 3580000, Chile; carobizama@gmail.com (C.B.); pagarmu@gmail.com (P.G.); 14 Department of Pathology, Hospital San Juan de Dios, Santiago 8320000, Chile; lorenagutierrezc@yahoo.es; 15 Institute of Medical Biometry, Universität Heidelberg, 69120 Heidelberg, Germany; lorenzo@imbi.uni-heidelberg.de; 16 Human Genetics Program, ICBM, Faculty of Medicine, Universidad de Chile, Santiago 8330015, Chile

**Keywords:** NGS-panel, target therapies, predictive biomarkers, somatic variants, gallbladder cancer, Latin America

## Abstract

Next-generation sequencing (NGS) is progressively being used in clinical practice. However, several barriers preclude using this technology for precision oncology in most Latin American countries. To overcome some of these barriers, we have designed a 25-gene panel that contains predictive biomarkers for most current and near-future available therapies in Chile and Latin America. Library preparation was optimized to account for low DNA integrity observed in formalin-fixed paraffin-embedded tissue. The workflow includes an automated bioinformatic pipeline that accounts for the underrepresentation of Latin Americans in genome databases. The panel detected small insertions, deletions, and single nucleotide variants down to allelic frequencies of 0.05 with high sensitivity, specificity, and reproducibility. The workflow was validated in 272 clinical samples from several solid tumor types, including gallbladder (GBC). More than 50 biomarkers were detected in these samples, mainly in BRCA1/2, KRAS, and PIK3CA genes. In GBC, biomarkers for PARP, EGFR, PIK3CA, mTOR, and Hedgehog signaling inhibitors were found. Thus, this small NGS panel is an accurate and sensitive method that may constitute a more cost-efficient alternative to multiple non-NGS assays and costly, large NGS panels. This kind of streamlined assay with automated bioinformatics analysis may facilitate the implementation of precision medicine in Latin America.

## 1. Introduction

In recent decades, molecular pathology has advanced substantially thanks to the exponential growth of genetic sequencing technology. The introduction of next-generation sequencing (NGS) opened the doors to high-throughput, multi-gene, massive data collection. This tool’s ability to sequence more, faster, and at a reduced cost has made it attractive for many clinical research applications. In cancer, using this technology to interrogate solid tumor samples has propelled a massive characterization of genes involved in the disease [1,2]. This rise in “oncogenomics” has been accompanied by an increase in cancer drug approval and development [3,4]. Identifying tumor-specific genetic signatures and correlating them to treatment outcome has evolved into a strategy termed “precision medicine”, a new diagnostic and treat process based on approved genomic biomarkers [4].

In Latin America and the Caribbean, 1.4 million new cancer cases were estimated to occur in 2018, while mortality rates varied among and within the region [5,6]. The most common types of cancer with the highest incidence are prostate (age standardized rate (ASR) 60.4), breast (ASR 56.8), colorectal (ASR 18.6), cervix uteri (ASR 15.2), lung (ASR 13.1), and stomach (ASR 9.5) cancers [1]. Overall, estimated age-standardized cancer incidence rates in Latin America are lower than those reported in North America and some European countries; however, the region exhibits higher mortality rates [7]. This paradox reflects the disparities in early diagnosis and treatment opportunities in the region.

In high- and medium-income countries, precision medicine is making its way into standard cancer treatment, improving survival and investigational drug trial success for many patients. A combination of factors prevents this helpful tool from becoming accessible to most of the world’s population. In Latin America, approved and available gene-based cancer screening assays are often solutions designed to meet first world standards. These large panels are great diagnostic tools in regions of abundant therapy options, but for Latin America and other regions, they are not cost effective, leaving behind a need for more comprehensive regional solutions. Additionally, the absence of automated clinician-ready reporting for many of these approved panels creates another major cost and obstacle to their widespread implementation. As a result, NGS-based oncology panels do not appear to be cost-effective solutions for many governments and are not being implemented in health and insurance systems despite local sequencing capabilities. This scenario creates an urgent need for customized validated solutions and data interpretation in a clinical environment [8].

In addition, an important caveat to interpreting Latin American cancer patient’s genetic data is the under-representation of Latin American individuals in global resources characterizing the frequency of both germinal and cancer genome variants. Great cancer genomics efforts, such as TCGA and ICGC, are deprived of minorities (including subjects of Hispanic ethnicity [9]), limiting their capacity to describe somatic mutations with a prevalence below 10% and overcome the somatic background mutation frequency in specific ethnic groups [10]. For instance, the average Amerindian ancestry in cancer patients across all cohorts in TCGA is about 4% [9,11]. Additionally, the Latin American population is under-represented in germline variant repositories, which may induce a false categorization and overestimation of somatic variants [12,13,14]. Thus, an additional blood sample should accompany the tumor sample, increasing the sequencing costs.

To address these challenges, we designed, optimized, and validated a hybridization-based target enrichment workflow with multiple automated analyses capable of detecting variants in 25 genes; 23 of them with an association to a drug’s response supported by the FDA or well-powered studies with consensus from experts in the field. Although this panel was designed to meet current and near-future Chilean precision oncology needs, we expect the panel and workflow to be relevant to other countries in the region. This workflow was locally validated using breast, colorectal, gastric, ovarian, pancreatic, and gallbladder tumor tissue samples and we reported its ability to detect single nucleotide variants (SNVs) and small insertions and deletions with high sensitivity and specificity. Additionally, high reproducibility was obtained for non-synonymous variants between and within runs. Finally, to address the shortage of health professionals trained in bioinformatics, the entire workflow, including quality control of sequencing data and calling for somatic variants, was automated and made available.

## 2. Materials and Methods

### 2.1. Panel Design

The panel targets hotspots, selected exons, or complete coding regions of 25 genes and includes predictive biomarkers in solid tumors. We refer to this panel, plus its associated workflow and analysis, as TumorSec™. For selecting targeted regions, biomarker genes classified with evidence 1, 2, 3a, 3b, R1, and R2 were selected for solid tumors in the OncoKB database (www.oncokb.org, accessed on 1 June 2021) [15]. Next, biomarker mutations with level of clinical evidence A, B, and C were selected in the Clinical Interpretation of Variants in Cancer, CiVic database (https://civicdb.org/home, accessed on 1 October 2018) [16] and the Variant Interpretation for Cancer Consortium (VICC) meta-knowledgebase [17]. Biomarkers were selected based on their level of evidence and incidence in the targeted tumor in Latin America. *TP53* and *ARID1A* complete coding regions were incorporated, as they contain prognosis and predictive chemotherapy biomarkers. The complete list of genes and drug associations is provided in Table 1.

Synthesis of the soluble, biotinylated probe library was performed on the NimbleGen cleavable array platform (SeqCap EZ Choice (RUO); Roche/NimbleGen, Basel, Switzerland). The probe design was optimized using the NimbleDesign software utility (NimbleGen, Roche, Basel, Switzerland).

### 2.2. Sample Information

In total, 183 tumor tissue samples were sequenced for this study. In total, 19 were freshly frozen (FF): 13 colorectal and 6 breast; 164 were formalin-fixed paraffin-embedded (FFPE) blocks: 9 breast, 71 ovary, 1 gastric, 43 gallbladder, and 40 colorectal tumors. Additionally, DNA from 89 whole blood or buffy coat samples were sequenced: 7 from colorectal and 72 from breast cancer patients. Colorectal and gastric cancer samples were obtained from the “Biobanco de Tejidos y Fluidos de la Universidad de Chile.” To capture real world heterogeneity in sample quality, breast, ovary, and gallbladder FFPE tissue samples were collected from the pathology services from several sites along the country (Fundación Arturo López Pérez, Clínica Dávila, Clínica Indisa, Red UC Christus, Biobanco de Tejidos y Fluidos, Hospital Padre Hurtado, Hospital Regional de Concepción, Hospital Regional de Talca, Hospital de Puerto Montt, Hospital San Juan de Dios, Hospital Santiago Oriente Doctor Luis Tisné Brousse, Instituto Nacional del Cáncer, Hospital del Salvador, Hospital Regional de Coquimbo, Hospital Regional de Arica, Hospital Clínico San Borja Arriarán).

### 2.3. Control Samples

Three reference standard DNA samples from Horizon Discovery (Cambridge, UK) were used as positive controls for variant calling: HD200 (FFPE somatic), HD793, and HD794 (germline BRCA1/2 variants).

### 2.4. DNA Extraction, Quantification, and Sample Quality Control

DNA from frozen tissues was extracted using the QIAamp DNA Mini Kit (Qiagen, Germantown, MD, USA). FFPE tissue DNA was extracted using GeneJet FFPE DNA Purification Kit and RecoverAll™ Total Nucleic Acid Isolation (Invitrogen, Thermo Fisher Scientific, Carslbad, CA, USA), following the manufacturer’s instructions, with overnight lysis instead of the suggested 1–2 h for FFPE tissue. Germline DNA was purified from whole blood samples or buffy coat using the Wizard^®^ Genomic DNA Purification Kit (Promega, Madison, WI, USA), according to manufacturer’s protocol.

Purified DNA was quantified using the Qubit(™) dsDNA HS Assay and Quant-IT(™) Picogreen^®^ dsDNA Reagent Kit (Invitrogen, Thermo Fisher Scientific, Carslbad, CA, USA). The purity of DNA was assessed by measuring the 260/280 nm absorbance ratio. For FFPE samples, fragment length and degradation were assessed using the HS Genomic DNA Analysis Kit (DNF-488) in a Fragment Analyzer (Agilent, formerly Advanced Analytical). DNA ranged from >1000 bp to 200 bp. Samples with <200 bp are not recommended for processing with the TumorSec workflow.

### 2.5. Library Preparation

Then, 100–150 ng of DNA (blood and frozen tissues) and 200 ng of DNA (FFPE) were used as input for sequencing library preparation. NGS libraries were prepared using KAPA HyperPlus Library Preparation Kit (Kapa Biosystems, Cape Town, South Africa). A double size selection was performed in libraries prepared with DNA from frozen tissue and blood and a single size selection for DNA from FFPE. Libraries were quantified using the QubitTM dsDNA HS Assay Kit (Invitrogen, Thermo Fisher Scientific, Carslbad, USA) and Quant-IT^TM^ Picogreen^®^ dsDNA Reagent Kit (Invitrogen). The quality of the amplified library was checked by measuring the 260/280 absorbance ratio and fragment’s length, using the HS NGS Analysis Kit (DNF-474) in a Fragment Analyzer (Agilent, formerly Advanced Analytical).

### 2.6. Target Enrichment

Prepared DNA libraries (1200 ng total mass) were captured by hybridization probes (Roche NimbleGen SeqCap EZ. Roche, Pleasanton, USA). The number of samples used for pre-capture multiplexing was based on sample type: six were pooled for FFPE and blood samples, while fresh frozen tumor samples were pooled in reactions of four. Captured libraries were assessed for concentration and size distribution to determine molarity.

### 2.7. Sequencing Run Set-Up

Libraries were diluted to a concentration of 4 nmol/L and processed for sequencing, according to the manufacturer’s instructions (Illumina, San Diego, CA, USA). The final captured library concentration for sequencing was 9.4–9.5 pM. Libraries were sequenced in an Illumina^®^ MiSeq System using paired-end, 300 cycles (MiSeq Reagent Kits v2, Illumina^®^ Illumina, San Diego, CA, USA).

### 2.8. Bioinformatic Analyses

The bioinformatic pipeline is summarized in Supplementary Figure S1. Filtering of reads and base correction were performed with the fastp v0.19.11 tool. The filtered reads align with the reference genome GRCh37/hg19 using Burrows–Wheeler Alignment mem (BWA mem v0.7.12). MarkDuplicates tool of Picard v2.20.2-8 was applied to identify duplicates. To reduce the number of mismatches to the reference genome, the reads were realigned with RealignerTargetCreator and IndelRealigner from GATK v3.8 [19]. Finally, the quality scores were re-calibrated with the combination of GATK’s BaseRecalibrator and PrintReads tools [19].

The SomaticSeq v.3.3.0 program was used to call the variants in single-mode using only tumor sequence data [20]. This tool maximizes the sensitivity by combining the result of five next-generation variant SNV callers—Mutect2 [21], VarScan2 [22], VarDict [23], LoFreq [24], and Strelka [25]—adding Scalpel for indels. The reported SNVs were identified by at least three out of five SNV callers, and the reported indels by at least three out of the six callers. The consensus variants obtained by SomatiSeq were annotated using the Cancer Genome Interpreter (https://www.cancergenomeinterpreter.org/, accessed on 1 June 2021) [26] and ANNOVAR [27] using RefGene, GnomAD v2.1.1 (genome and exome), ESP6500, ExAC v0.3, 1000 Genomes phase 3, CADD v1.3, dbSNP v150, COSMIC v92, and CLINVAR.

### 2.9. Variant Filtering and Sequence Quality Reporting

Variants with allele frequencies greater than 0.5 and with an altered allele depth ≥12 reads were selected. These thresholds were established as the limit of detection (LOD) for the NGS TumorSec panel following the recommendations of the Association for Molecular Pathology and College of American Pathologists [28]. Polymorphisms were eliminated, discarding alleles reported in 1000 Genomes, ESP6500, GnomAD, or ExAC [27] with a frequency greater than 0.01. Filtering was extended to include all under-represented populations that had information in GnomAD and ExAC. Additionally, a dataset containing genetic variants in Chilean individuals was used for further filtering.

The bioinformatics pipeline was executed automatically, creating pdf reports that allowed an easy view of quality metrics per sample. For this purpose, the programs FastQC v0.11.8, Qualimap v2.2.2a, Mosdepth v0.2.5, and MultiQC v1.8 were executed between the pre-processing of the bioinformatics workflow. The main metrics are the number of initial raw reads, the percentage of filtered reads, the duplication rate, the number of reads on target regions, the average depth on-target regions, the uniformity percentage, and the ratio of on-target regions with a minimum coverage of 100× to 500×. For variant calling and annotation, the coverage threshold for FFPE and FF was set at 300× in at least 80% of target regions.

The bioinformatic pipeline and tutorial can be found in the GitHub repository called Pipeline-TumorSec (https://github.com/u-genoma/Pipeline-TumorSec, accessed on 1 June 2021).

### 2.10. Germline Variant Calling

Data were pre-processed following the pipeline shown in Supplementary Figure S1. Variant calls were made using the GATK HaplotypeCaller tool. A minimum confidence threshold of 30 was set for variant calling. Additionally, a coverage threshold was set at 200× in at least 80% of target regions. Finally, a variant calling hard-filter for SNPs and indels was applied separately following GATK recommendations [29].

## 3. Results

### 3.1. Panel Design and Sequencing Metrics

A total of 25 genes were included in this target enrichment panel, covering 98 kb of sequence length. Design details broken down by gene are shown in Table 1. In total, 79% (15/19) of fresh frozen, 71% (116/164) of FFPE, and 89% (79/89) of blood samples processed passed the sequencing quality threshold, capturing a minimum of 80% of target regions at a depth of 300×. A summary of relevant sequencing metrics for all 210 passed samples is shown in Supplementary Table S1. FFPE samples showed a high percentage of duplicates and off-target reads. Uniformity was >90% for all sample types and >90% of targeted regions had ≥300× coverage (Supplementary Figure S2).

### 3.2. Panel Performance

**Figure 1:**
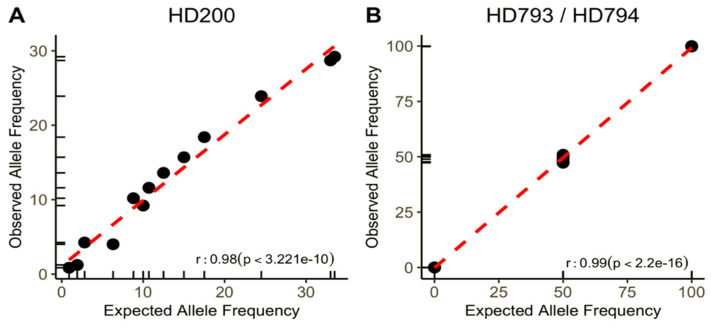
Performance and analytical sensitivity of the panel. Correlation between expected allelic frequencies for reported variants in commercial standard control samples and those observed by the assay: (**A**) FFPE Horizon Discovery sample (HD 200); (**B**) germline DNA samples with BRCA1/2 mutations (HD 793 and HD 794).

The panel’s performance was calculated using the reference HD200 (Horizon Discovery) standard FFPE sample containing characterized mutations in the following genes: *BRAF, KIT, EGFR, KRAS, NRAS, PIK3CA, ARID1A,* and *BRCA2*. As observed in Figure 1A, the assay captured all 13 positive variants. A 0.98 coefficient of correlation (r-squared) was extrapolated with a *p*-value of 3.221 × 10^−10^ between expected variant allele frequencies (VAF) from the positive control and those reported by the assay. VAFs ranged from 24.5% to as low as 1%, showing the assay’s high analytical sensitivity.

Additionally, the panel’s performance for detecting *BRCA1/2* germline mutations, which are predictive biomarkers for PARP inhibitor therapy in breast, ovarian, and prostate cancer, was tested (Table 1). Thus, references DNA HD793 and HD794 (Horizon Discovery), which contain known germline variants in *BRCA1* and *BRCA2* at VAF of 50 and 100%, were sequenced. Figure 1B shows that 11 out of 11 reported variants were detected at the expected VAF. The correlation coefficient between expected and reported VAF is 0.99 with a *p*-value of 2.2 × 10^−16^. Importantly, no mutations were detected in the 15 positions reported as “no-mutated” (true negatives), showing the assay’s high specificity.

**Figure 2:**
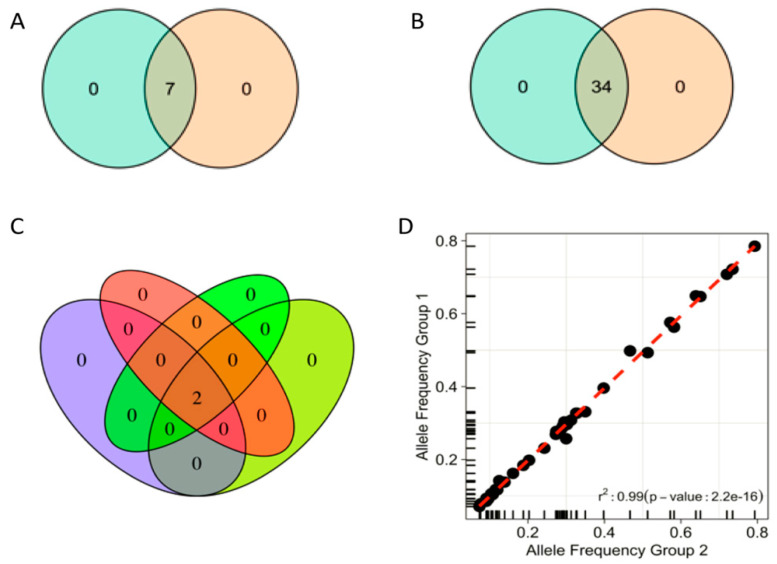
Reproducibility and repeatability of the assay: (**A**) within sequencing run reproducibility based on three patient samples, each from a different tumor type (colon, ovaries, gallbladder). Venn diagram displays variants observed in two different library preparations for each of the three samples. (**B**) Repeatability between sequencing runs was assessed using the same libraries from three different FFPE cancer and control (HD200) samples in two separate sequencing runs. Venn diagram displays variants observed between two sequencing runs. (**C**) One ovarian cancer sample was processed using four different combinations (colors) of library preparation and sequencing runs. (**D**) Correlation between allele frequencies of variants obtained in repeatability and reproducibility tests (displayed in (**A**,**B**)).

For reproducibility assessment, three FFPE samples from different tumors (colorectal, ovary, and gallbladder) were used to prepare two separate libraries each. All samples passed the sequencing metrics threshold with ≥300× coverage in 99.6% of target regions and 89% uniformity. A 100% concordance among non-synonymous variants detected in the different libraries was observed (Figure 2A).

Inter-runs repeatability was assessed using four FFPE samples (three ovaries and one HD control). Different libraries were sequenced in different runs. Reproducibility of sequencing metrics (94% of target regions with ≥300× coverage and ≥87% uniformity) and concordance of detected variants were also observed (Figure 2B). One ovarian FFPE sample was assessed in three different library preparations and three separate sequencing runs (Figure 2C). A high correlation (r = 0.99) was observed between VAFs called in all the different settings (Figure 2D).

### 3.3. Comparison between FFPE, Fresh Frozen, and Blood gDNA

To assess whether the protocol and bioinformatic workflow for detecting somatic mutations discard FFPE-induced artifacts and germline variants, DNA from FFPE, fresh frozen (FF) tumor, and buffy coat samples from six ductal breast carcinoma subjects was sequenced.

**Figure 3:**
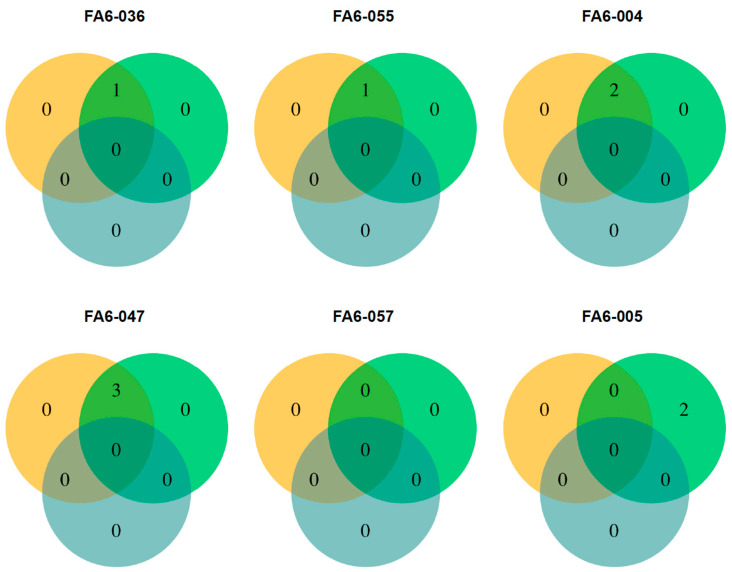
Mutations found in FFPE and fresh frozen tumor tissue, and blood from same individuals. Venn diagram outlining non-synonymous variants reported for six different patients diagnosed with ductal breast carcinoma among three different sample types for each: FFPE tumor (orange), fresh frozen tumor (green), and buffy coat (light blue).

Variants detected among all sample triads for each of the six subjects are outlined in Figure 3. Nine variants in the FF sample set were reported. Seven of these variants were also reported in the matching FFPE samples. It is worth noting that the two variants detected in an FF sample (FA6-005) were found in the FFPE sample but at frequencies <5% (the LOD established for the assay).

**Figure 4:**
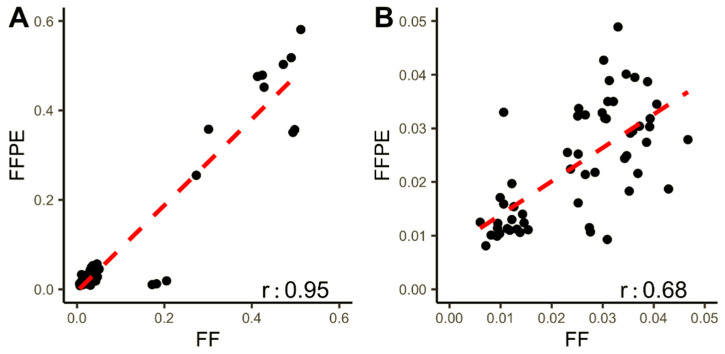
Correlation between allele frequencies in synonymous and non-synonymous variants found in paired FFPE and fresh frozen samples: (**A**) correlation between variant allele frequencies (VAF) in all variants found in FFPE and fresh frozen samples (FF). (**B**) Correlation between frequencies of variants found at VAF below LOD (0.05).

To further explore FF and FFPE samples’ concordance, the allele frequencies of both synonymous and non-synonymous variants detected in both sample types were plotted (Figure 4). Variants with AF < 5% display a low r-value (0.68, *p*-value of 2.479 × 10^−9^). However, when all variants (73) are analyzed, correlation increases (r = 0.95, *p*-value of < 2.2 × 10^−16^). Importantly, no germline variants and no variants exclusive for FFPE samples were detected using the pipeline for somatic mutations.

### 3.4. Validation of the Assay in Clinical Samples

To validate the assay and analysis capabilities in “real world” samples, 183 tumor biopsies from different clinical sites were processed. In total, 131 out of the 183 were successfully sequenced (116 FFPE and 15 FF): breast (14), ovary (69), gastric (1), gallbladder (31), and colorectal (16).

All variants with allelic frequency > 0.01 reported in at least one of the following four germline population variant’s databases (PVDs): GnomAD, ESP6500, ExAC, and 1000 Genomes, were eliminated. However, given that the Latin American population is not well represented in these repositories, somatic mutations were initially overestimated due to their absence or low (<1%) VAF. Thus, the classification of remaining variants was performed using the algorithm depicted in Supplementary Figure S2. This algorithm was designed based on recommendations from Sukhai et al. (2019) [30] and using annotations in COSMIC, dbSNP, CLINVAR, and PVDs databases. Filtering was extended to include all under-represented populations that have information in GnomAD and ExAC. Additionally, a local genetic germline database from Chilean individuals was used. The resulting variants were classified as germline, somatic, putative germline, putative somatic, putative germline novel, or putative somatic novel.

**Figure 5:**
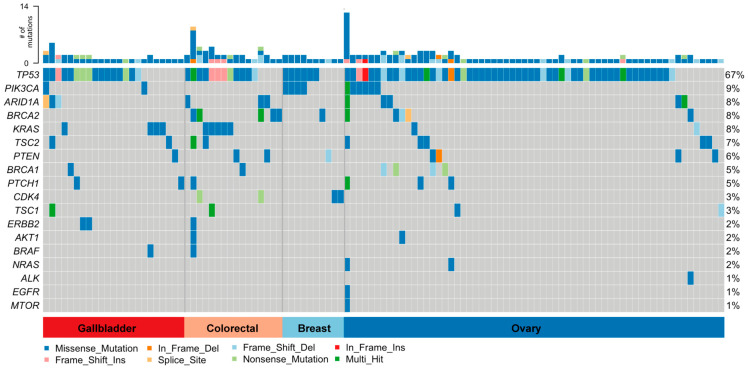
Non-synonymous somatic variants detected across all tumor samples; 197 non-synonymous somatic variants were detected in 111 out of 131 gallbladder, colorectal, breast, and ovary cancer samples.

A total of 256 protein-affecting variants were found in the 131 samples. Among these, 197 non-synonymous somatic and putative known and novel somatic variants were identified in 111 out of the 131 samples (85%) (Table 2); moreover, 144 were unique variants. Figure 5 shows a breakdown of somatic mutations by gene and tumor type. Non-synonymous variants are shown according to mutation type (missense and nonsense mutations, frameshift causing deletions and insertions, and variants positioned in splice sites). Overall, TP53, BRCA2, PIK3CA, ARID1A, KRAS, TSC2, PTEN, and BRCA1 were the most frequently mutated genes. TP53 and PIK3CA were the most prevalent mutations in ovary cancer; TP53, BRCA2, and KRAS in colorectal cancer; and TP53 and PIK3CA in breast cancer. In 31 samples of GBC, we found 36 mutations in TP53 (16), KRAS (4), ARID1A (3), ERBB2 (2), TSC2 (2), PIK3CA (2), PTCH1 (2), TSC1 (2), and BRCA1, PTEN, and BRAF (1 each).

### 3.5. Identification of Biomarkers for Targeted Therapies

**Figure 6:**
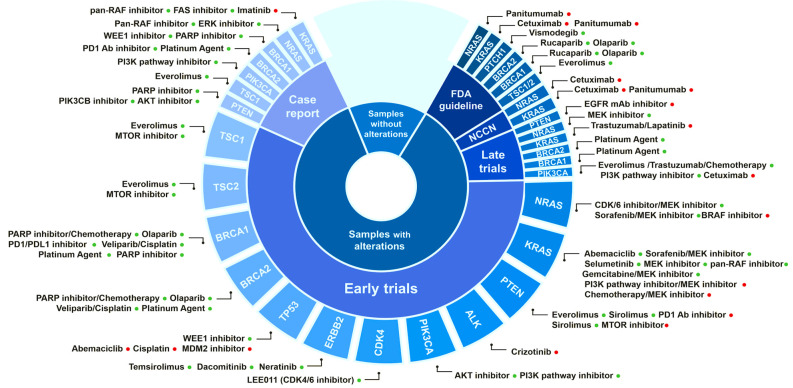
Predictive biomarkers for therapy response identified in the analyzed clinical tumor samples. The biomarker gene, the associated drugs, and the level of evidence supporting this association in solid tumors are depicted. Positive (drug-responsive) and negative (drug-resistant) associations are indicated by green and red dots, respectively. Information on these interactions was obtained from “The Cancer Genome Interpreter” [26].

In total, 137 (69.5%) out of the 197 identified somatic variants are described as a biomarker for drug response, which is supported by different levels of evidence: FDA guidelines (44) and clinical guidelines (9), late trials (37), early trials (119), case report (42), and (113) pre-clinical data. The affected gene and target drug associations with supporting evidence from “case reports” to “FDA guidelines” are depicted in Figure 6, which outlines: (1) the fraction of samples with reported genetic alterations; (2) the level of existing evidence for the biomarker; (3) the gene affected; and (4) the drug association (resistant or responsive). Table 3 contains a detailed description of the biomarker mutations supported by FDA and NCCN clinical guidelines found in this study in all samples.

In GBC samples, we found biomarker mutations in eight genes, with supporting evidence ranging from case reports to FDA and NCCN guidelines in different tumor types (Table 4). It is worth highlighting the presence of predictive biomarkers for drugs that are currently in use for treating different cancers, such as PARP, ERBB2, EGFR, PIK3CA, mTOR, and Hedgehog signaling inhibitors.

## 4. Discussion

As a result of its global adoption and implementation, the clinical utility of NGS in the field of oncology has a rapidly growing body of evidence. The ability to obtain massive amounts of genetic information from small amounts of tissue provides clear advantages for decision-making processes against cancer [2,4,31]. Nevertheless, a large portion of cancer patients around the world do not have this option readily available. This work attempts to favor the implementation of NGS in the Latin American health system by showcasing a locally developed assay, accompanied by an open-source automated analysis focused on the target population’s needs.

A critical consideration for implementing NGS in low resource settings is finding a workflow compatible with low-quality, highly degraded samples. Although fresh frozen tissue is the gold standard for molecular analyses, its use in clinical practice is impractical because of its high cost and technical difficulties related to its obtainment, processing, and storing. The sample storage infrastructure found in the developed world, with dedicated −80 °C and −20 °C freezers, is too often not in the budget for many Latin American diagnostic laboratories. FFPE tissue samples are much more cost effective, as they can be stored at room temperature. However, tumor biopsies in this region are often fixed with formalin with different protocols and laboratory environments, producing varied DNA damage during and after the formalin fixation process (e.g., fragmentation, degradation, crosslinking [32,33]).

DNA quality is affected by the type of formalin used for tissue fixation and the time since preservation [34], both of which vary highly in laboratories across Chile. A total of 48 FFPE samples failed to pass the DNA, library, or sequencing quality controls. Most of the failed samples were colorectal (33/40) and gallbladder (12/43) samples. Twenty-four failed colorectal samples had low on-target rates, suggesting issues with hybridization and/or capture, while the 12 gallbladder samples did not pass the library preparation quality metrics.

In general, FFPE samples showed a higher percentage of duplicates and off-target reads (Table S1). However, these characteristics do not affect sequencing results. FFPE samples have the highest mean on-target region’s coverage compared to FF and BC. Removing duplicates is intended to reduce noise during the variant identification process and minimize false positives. These results suggest removing duplicates has little effect on this panel’s performance at the sequencing depths of interest (~300×). As sequencing technologies continue to advance, PCR duplicate removal will become less of an issue [35].

Somatic mutation analysis and fusion detection are critical in cancer research [1,15]. In this project, we focused on creating a cost-effective regional tool with clinical relevance. As a result of our approach, a tailored DNA based panel, we lack the ability to detect relevant RNA gene alterations such as fusions. Although side-by-side DNA and RNA analysis would provide a more complete understanding of gene alteration profiles, it can be costly and time consuming. Future solutions such as total nucleic acid library preparation followed by target enrichment could provide a more comprehensive analysis in one workflow. In the meantime, this panel detects point mutations in ALK, MET, and ROS1, which have been associated with resistance to TKI.

Currently, there is no community consensus about the most appropriate variant caller for somatic mutations [36]. For this reason, the bioinformatic pipeline for variant calling used in this work incorporated six variant callers capable of producing highly accurate somatic mutation calls for both SNVs and small indels. Somatic variant callers discard germline variants by interrogating the reference genomes and population databases, such as 1000 Genomes, where Latin American genetic variation is not well represented. Thus, we implemented a more accurate bioinformatic pipeline that allowed variants’ classification as somatic, germline, and putative somatic/germline. This variant calling process highlights the extra layer of difficulty Latin American researchers and clinical laboratories face due to the absence of reference genomes representative of our population in the major databases. The overestimation of somatic variants is a problem when facing the tumor of a patient from any region or ancestry without a reference genome informative of the genetic variation in that specific population. This issue is critical for therapy determinants, such as tumor mutational load, which should be carefully interpreted in these patients [37].

The addition of reference genomes representative of the population in combination with the genetic characterization of more cancers will help address questions involving the distribution of genetic alterations among different world populations. 

The best approach for resolving the somatic vs. germline mutations issue is to include respective blood samples alongside biopsies. However, this raises the assay’s cost per patient, which may delay the assay’s implementation.

To achieve more accurate somatic variant calling, further efforts towards genetic characterization of the Latin American and other under-studied populations are needed. Building an inclusive tumor reference genome database will allow for the discovery of novel somatic mutations and non-explored correlations to the disease.

The assay was designed to meet the biomarker needs in countries with low participation in clinical trials and in which a limited number of drugs are available or currently in use in clinical practice. Among the 131 predictive biomarkers for therapies response detected by the assay, 52 are supported by evidence recognized by the FDA and NCCN clinical guidelines.

Although a small number of GBC samples was successfully sequenced, biomarker mutations in nine genes were identified. Importantly, these are predictive biomarkers for drugs that are currently in use for treating different cancers, such as PARP, ERBB2, EGFR, PIK3CA, mTOR, and Hedgehog signaling inhibitors. Since most of these drugs are available in Chile and LATAM, finding predictive biomarkers in GBC generates opportunities for specific and basket clinical trials including GBC patients from Chile and other regions in LATAM, where this disease has an unusually high prevalence.

## Figures and Tables

**Table 1:** Genes included in the panel and their therapy association.

Gene	Drugs	Tumor Type	Evidence ^1^
AKT1 *	AZD-5363	Breast cancer Ovarian cancer Endometrial cancer	B
ALK	Ceritinib Crizotinib Alectinib Brigatinib Lorlatinib	Non-small cell lung cancer	A
ARID1A *	Trastuzumab ENMD-2076 Bevacizumab Everolimus	Breast cancer Ovarian clear cell cancer Renal cell carcinoma	C
BRAF *	Encorafenib + Cetuximab Vemurafenib Dabrafenib Trametinib + Dabrafenib Cobimetinib + Vemurafenib Trametinib Encorafenib + Binimetinib Vemurafenib + Cobimetinib, Trametinib + Dabrafenib Vemurafenib + Cobimetinib Encorafenib + Panitumumab	Colorectal cancer Melanoma Non-small cell lung cancer Anaplastic thyroid cancer Hairy cell leukemia Pilocytic astrocytoma Ganglioglioma Pleomorphic xanthoastrocytoma	A
BRCA1 *	Olaparib Niraparib Rucaparib Talazoparib	Ovarian cancer Peritoneal serous carcinoma Breast cancer Prostate cancer Ovary/fallopian tube Pancreatic cancer	A
BRCA2 *	Olaparib Rucaparib Talazoparib	Ovarian cancer Peritoneal serous carcinoma Breast cancer Prostate cancer Ovary/fallopian tube	A
CDK4 *	Palbociclib Abemaciclib	Liposarcoma	B
EGFR	Erlotinib Afatinib Osimertinib Gefitinib Dacomitinib	Non-small cell lung cancer	A
ERBB2	Trastuzumab Fam-Trastuzumab deruxtecan-nxki Trastuzumab + Pembrolizumab Afatinib	Breast cancer Gastric adenocarcinoma Gastroesophageal junction adenocarcinoma Non-small cell lung cancer	A
ESR1	Anastrozole Fulvestrant Palbociclib	Breast cancer	B
IDH2	Enasidenib	Acute myeloid leukemia	A
KIT	Sunitinib Imatinib Regorafenib Sorafenib Ripretinib	Gastrointestinal stromal tumor Melanoma	A
KRAS *	Cetuximab Panitumumab Erlotinib Lapatinib Regorafenib Selumetinib Gefitinib Afatinib Icotinib Irinotecan	Colorectal cancer Non-small cell lung cancer	A
MET	Crizotinib Capmatinib Tepotinib	Non-small cell lung cancer	A
MTOR *	Everolimus Temsirolimus	Renal cell carcinoma Bladder Cancer	B
NRAS *	Cetuximab Panitumumab	Colorectal cancer	A
PDGFRA	Imatinib Sunitinib Regorafenib	Gastrointestinal stromal tumor	A
PI3KCA *	Buparlisib Serabelisib Alpelisib Copanlisib	Breast cancer	A
PTCH1 *	Vismodegib	Skin basal cell carcinoma Squamous cell carcinoma Medulloblastoma	A
PTEN *	Everolimus Pembrolizumab Cetuximab Sorafenib	Renal cell carcinoma Glioma Head and neck squamous cell carcinoma Colorectal cancer Hepatocellular carcinoma	B
ROS1	Crizotinib Alectinib Ceritinib	Non-small cell lung cancer	C
SMO	Vismodegib	Skin basal cell carcinoma	B
TP53 *	Prognosis	Various	A
TSC1 *	Everolimus	Giant cell astrocytoma Renal cell carcinoma Renal angiomyolipoma	A
TSC2 *	MTOR inhibitors	Giant cell astrocytoma Renal cell carcinoma Renal angiomyolipoma	A

^1^ Level of evidence according to AMP/ASCO/CAP Level A: FDA-approved therapy included in professional guidelines; B: well-powered studies with consensus from experts in the field; C: FDA-approved therapies for different tumor types or investigational therapies [18]. The highest level of evidence is shown. Some biomarkers may have additional indications with lower levels of evidence for different cancer types or protocols. * For these genes, all exons were targeted.

**Table 2:** Classification of non-synonymous variants found in 131 quality-passed tumor samples. The number of total and unique variants by classification is shown.

Classification of Variants	Total Variants	Unique Variants
Germline	55	26
Putative Novel Germline	4	3
Somatic	125	86
Putative Somatic	13	13
Putative Novel Somatic	59	45
**Total**	**256**	**173**

**Table 3:** Biomarker mutations supported by FDA (*n* = 44) and NCCN guidelines (*n* = 9), found in the analyzed clinical samples. The associated drug and the mutation’s predictive effect are indicated.

Gene	Mutation	Drug	Effect
BRCA1	E1609 *, L702Wfs * 5, N1745Tfs * 20, Q1273 *, V370I	Rucaparib (PARP inhibitor) Olaparib (PARP inhibitor)	Responsive
BRCA2	A2603S, D1796Mfs * 9, K3327Nfs * 13, L1114V, splice_acceptor_variant, T2783Afs * 13, T2790I, I1364M, L398P, D635G, R2034C		
KRAS	A146V, Q61H G12A, G12D, G12V, L19F, Q25 * fs * 1	Panitumumab (EGFR mAb inhibitor) Cetuximab (EGFR mAb inhibitor)	Resistant
NRAS	G12C, Q61R	Panitumumab (EGFR mAb inhibitor) Cetuximab (EGFR mAb inhibitor)	Resistant
PIK3CA	H1047R, E545A, E545K, E542K, R88Q, N345S, E579K	Alpesilib + Fulvestrant	Responsive
PTCH1	R441H, D717N, H1240R, P725S, V580A, T677A, N871D	Vismodegib (SHH inhibitor)	Responsive
TSC1	K375Sfs * 30, L826Q, L827Q, T582S	Everolimus (MTOR inhibitor)	Responsive
TSC2	R1729C, S1530L, K533delK, A460T, A950T, D1084G, P1771L, S1096C, T154I		

*: It is a mutation nomenclature.

**Table 4:** Biomarker mutations found in GBC samples. The number of samples with the mutation, associated drug, supporting level of evidence, and tumor where the evidence was generated are indicated.

# of Samples	Gene	Mutation	Drugs	Evidence	Tumor Tested
1	BRCA1	V370I	Rucaparib (PARP inhibitor) Olaparib (PARP inhibitor) WEE1 inhibitor Platinum agent (chemotherapy) Veliparib; Cisplatin (PARP inhibitor; chemotherapy)	FDA guidelines Case report Early trials	OV BRCA BRCA OV OV
2	ERBB2	L755S	Dacomitinib (Pan ERBB inhibitor) Neratinib (ERBB2 inhibitor) Temsirolimus (MTOR inhibitor)	Early trials	NSCLC CANCER, LUAD
4	KRAS	G12A G12V Q61H G12D	Panitumumab (EGFR mAb inhibitor) Cetuximab (EGFR mAb inhibitor) Trastuzumab; Lapatinib (ERBB2 mAb inhibitor; ERBB2 inhibitor) Gemcitabine; MEK inhibitor (chemotherapy; MEK inhibitor) MEK inhibitor Selumetinib (MEK inhibitor) PI3K pathway inhibitor; MEK inhibitor Abemaciclib (CDK4/6 inhibitor) Imatinib (BCR-ABL inhibitor and KIT inhibitor)	FDA guidelines FDA guidelines Late trials Early trials Early trials Early trials Early trials Early trials Case report	COREAD LUAD PA NSCLC, HC, BT, L L PA L L GIST
1	PIK3CA	E545K	PI3K pathway inhibitor Everolimus; Trastuzumab; chemotherapy (MTOR inhibitor; ERBB2 mAb inhibitor; chemotherapy) Cetuximab (EGFR mAb inhibitor) AKT inhibitor PI3K pathway inhibitor PI3K pathway inhibitor	FDA guidelines Late trials Late trials Early trials Early trials Case report	BRCA BRCA COREAD BRCA ED, OV, CESC BLCA, HNSC, L
1	PTCH1	P725S	Vismodegib (SHH inhibitor)	FDA guidelines	BCC, MB
11	TP53	E171 * G244S G266V K321Ifs * 10 L257P R280T V173Gfs * 10 R213 * R248W R273C W53 * R273H R248Q Q192 * C238F	MDM2 inhibitor Abemaciclib (CDK4/6 inhibitor) Cisplatin (chemotherapy) WEE1 inhibitor	Early trials Early trials Early trials Early trials	LIP BRCA FGCT, MGCT OV
1	TSC1	L826Q	Everolimus (MTOR inhibitor)	FDA guidelines Early trials Case report	GCA, RA BLCA ST, S, R
2	TSC2	D1084G S1096C	Everolimus (MTOR inhibitor)	FDA guidelines	GCA, RA
1	ARID1A ^#^	Splice acceptor variant	(EZH2 inhibitor) (PD1 inhibitor) (PARP inhibitor) (ATR inhibitor)	Pre-clinical Pre-clinical Pre-clinical Pre-clinical	OV OV CANCER CANCER

^#^ For ARID1A, pre-clinical evidence is shown, *: It is a mutation nomenclature.

## Data Availability

The bioinformatic pipeline and tutorial can be found in the GitHub repository called Pipeline-TumorSec (https://github.com/u-genoma/Pipeline-TumorSec).

## Supplementary Materials

The following are available online at https://www.mdpi.com/article/10.3390/jpm11090899/s1, Table S1: Sequencing metrics (median) for all samples that passed QC filters. Figure S1: General workflow of the bioinformatic pipeline for identifying somatic variants. Figure S2: Sequencing metrics and mean coverage of targeted genes according to sample type. Figure S3: Algorithm for variant´s classification.
