# Supplementary material for: Validation of an NGS Panel Designed for Detection of Actionable Mutations in Tumors Common in Latin America
Source: J Pers Med. 2021 Sep 8;11(9):899. doi: 10.3390/jpm11090899 (PMC8472524; doi:10.3390/jpm11090899)
Supplement: Supplementary file 1 [file jpm-11-00899-s001.zip › jpm-1311481-supplementary.pdf]

Supplementary Materials.

Table S1. Sequencing metrics (median) for all samples that passed QC.

	Sample Type		
	**FFPE (n=116)**	**Blood (n=79)**	**Fresh Frozen (n=15)**
% Duplicated reads	34.8	9.5	9.15
% of reads in target region	59.1	69.1	65.8
Average coverage in target region	1,145.5	798.5	789.1
% Uniformity	94.1	96.0	91.1
% of target regions with coverage o 300X	99.0	97.4	90.0

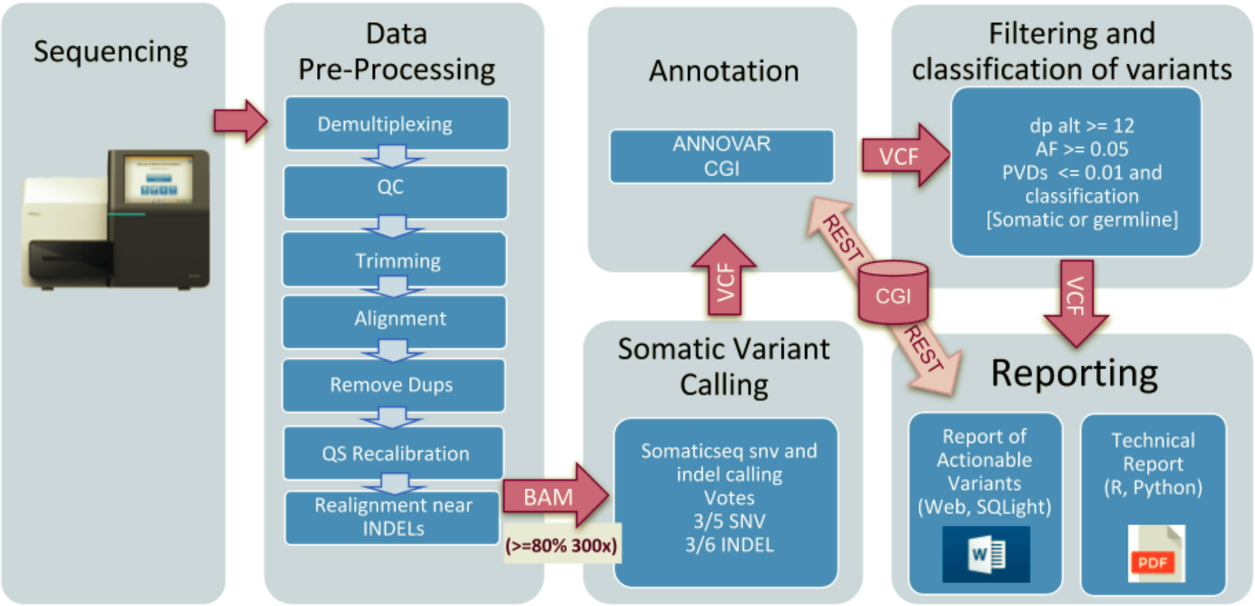

Figure S1. General workflow of the bioinformatic pipeline for identifying somatic variants. Gray boxes correspond to automated processes. Blue squares show the main sub-processes. Key input and output files are shown in arrows.

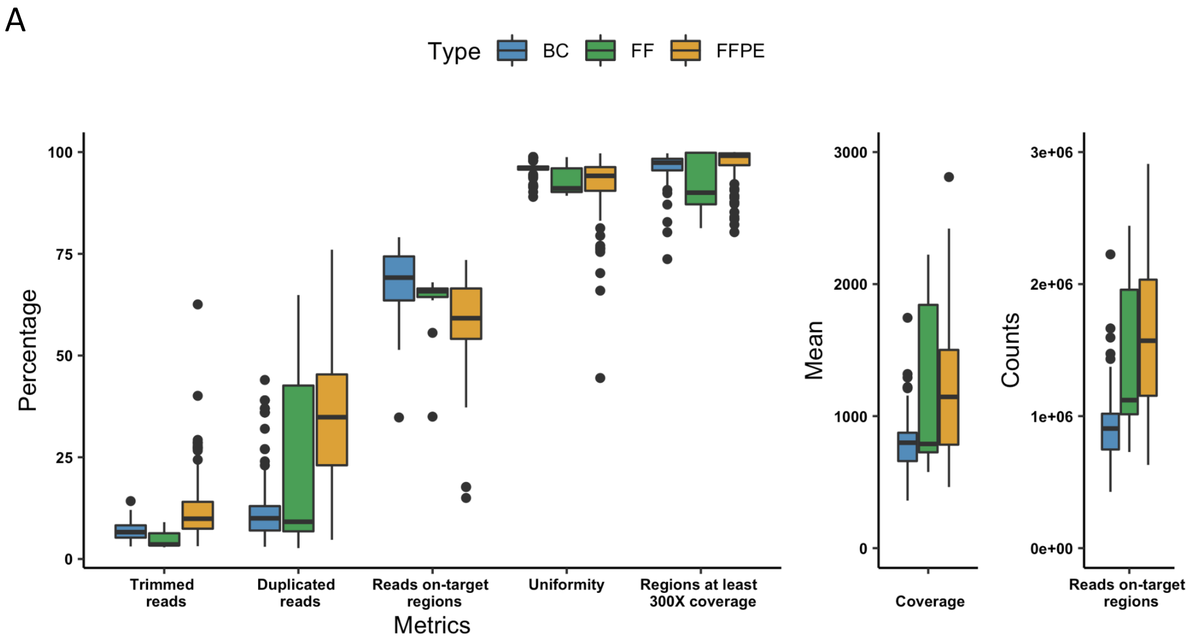

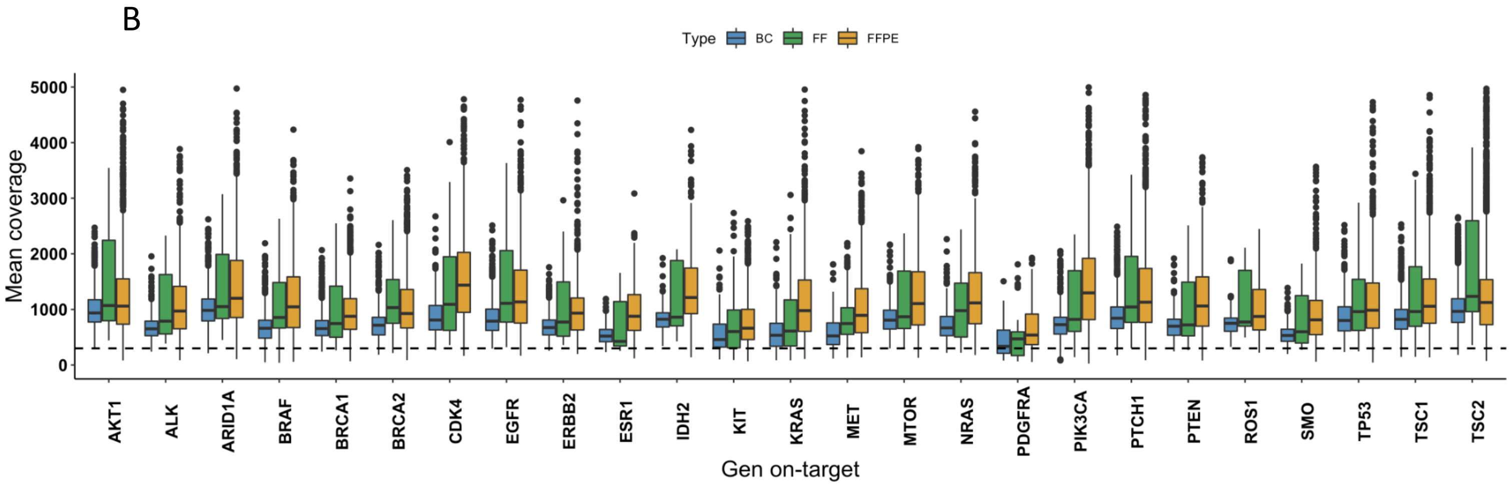

Figure S2. Sequencing metrics and mean coverage of targeted genes according to sample type A) Box plot of sequencing metrics by type of sample (79 BC, 15 FF, and 116 FFPE). BC: Buffy coat; FF: Fresh Frozen; FFPE: Formalix-fix Paraffin-Embedded. Each box shows the data distribution divided into quartiles. The centerline shows the median, and outlier points are in black. **B)** Box plot of mean coverage of on-target regions per-gene for each type of sample: BC (blue), FF (green), and FFPE (orange). The centerline shows the median, and outlier points are shown in black. The vertical dotted line shows 300X coverage.

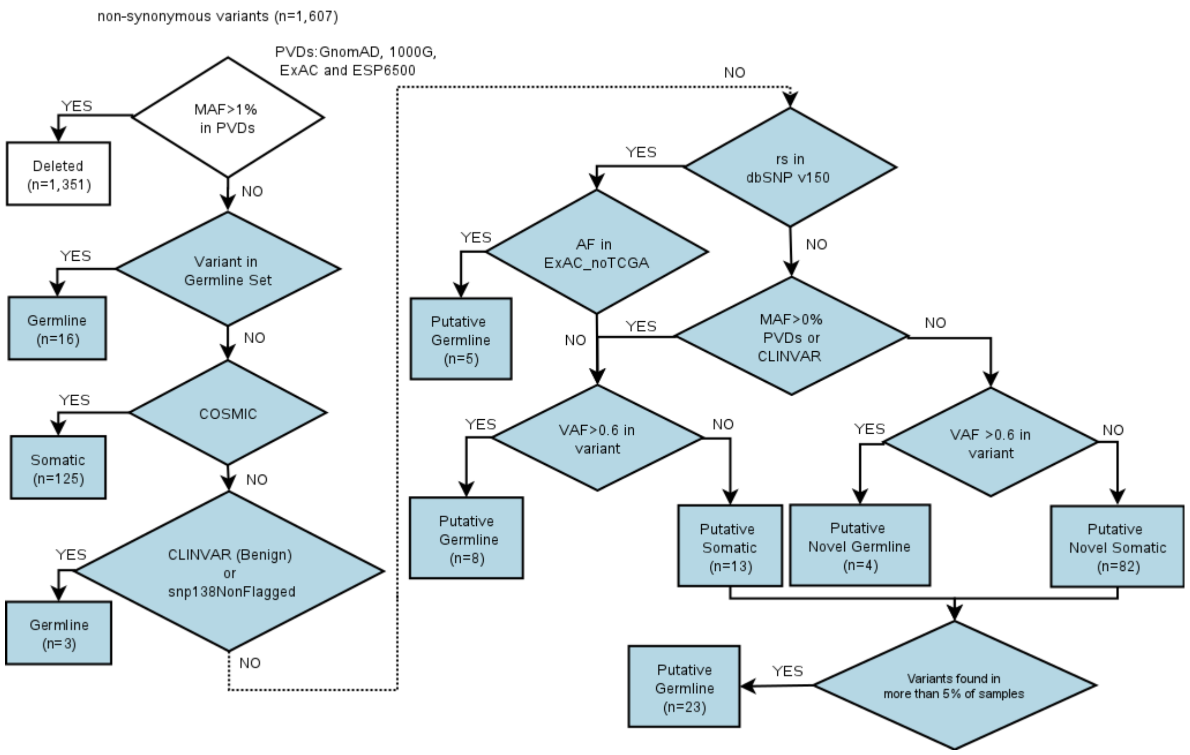

Figure S3. Algorithm for variant´s classification. Each conditional sequentially classifies variants as somatic or germinal (known, putative, or putative novel). 1) Variants with MAF over 1% in PVDs are deleted (n=1,351). 2) variants found in blood samples (BC) are labeled as germline in tumor samples (n=16). 3) Variants with a COSMIC annotation are labeled as somatic (n=125). 4) Benign or likely benign variants according to Clinvar or rs in snp138NonFlaged, are labeled as putative germline (n=3). 5) Variants with rs in dbSNP and reported in ExAC no-TCGA are labeled as putative germline (n=5). 6-7) Variants without classification and rs in dbSNP, MAF between 0-1% in PVDs or Clinvar information, are labeled as putative germline (n=8) or putative somatic (n=13) when VAF in the samples is >60% or <60%, respectively. 8) Variants without classification are labeled as putative novel germline (n=4) or putative novel somatic (n=82) when VAF in the samples is >60% or <60%, respectively. 9) Finally, variants classified as putative somatic or putative novel somatic found in more than 3 (5%) samples are re-classified as putative germline (n=23). MAF: minor allele frequency, PVDs: Population Variants Databases, COSMIC: Catalogue of Somatic Mutations in Cancer, Snp138NonFlaged: dbSNP without flagged SNPs (SNPs with MAF < 1% or unknown, mapping only once to reference assembly or with a clinical association, rs: Reference SNP, VAF: Variant Allele Frequency.
